# Adherence to antiretrovirals in people coinfected with the human immunodeficiency virus and tuberculosis^1^

**DOI:** 10.1590/1518-8345.0537.2691

**Published:** 2016-05-17

**Authors:** Larissa de Araújo Lemos, Maria Luciana Teles Fiuza, Renata Karina Reis, André Carvalho Ferrer, Elucir Gir, Marli Teresinha Gimeniz Galvão

**Affiliations:** 2 Doctoral Student, Departamento de Enfermagem, Universidade Federal do Ceará, Fortaleza, CE, Brazil. RN, Instituto de Saúde e Gestão Hospitalar, Fortaleza, CE, Brazil; 3 Doctoral Student, Departamento de Enfermagem, Universidade Federal do Ceará, Fortaleza, CE, Brazil. RN, Hospital Universitário Walter Cantídeo, Fortaleza, CE, Brazil; 4 PhD, Professor, Escola de Enfermagem de Ribeirão Preto, Universidade de São Paulo, PAHO/WHO Collaborating Centre for Nursing Research Development, Ribeirão Preto, SP, Brazil; 5 PhD, Professor, Faculdade de Medicina, Universidade Federal do Ceará, Fortaleza, CE, Brazil; 6 PhD, Full Professor, Escola de Enfermagem de Ribeirão Preto, Universidade de São Paulo, PAHO/WHO Collaborating Centre for Nursing Research Development, Ribeirão Preto, SP, Brazil; 7 PhD, Professor, Departamento de Enfermagem, Universidade Federal do Ceará, Fortaleza, CE, Brazil

**Keywords:** Medication Adherence, Coinfection, HIV, Tuberculosis

## Abstract

**Objective:**

assess the adherence levels to antiretroviral therapy in people coinfected with HIV/tuberculosis and correlate these levels with the sociodemographic and clinical variables of the study population.

**Method:**

cross-sectional study involving 74 male and female adults coinfected with HIV/tuberculosis. For the data collection, a sociodemographic and clinical assessment form and the Antiretroviral Treatment Adherence Assessment Questionnaire were used. For the data analysis, the software STATA version 11 was used, through descriptive statistics, Fisher's chi-square exact test and the probability test.

**Results:**

men were predominant (79.7%), between 30 and 39 years of age (35.1%), low income (75.7%) and pulmonary tuberculosis (71.6%). Adherence to antiretroviral therapy was inappropriate in 78.1% of the men; 61.0% of single people; 47.0% unemployed and 76.5% among people gaining less than one minimum wage. A significant difference was observed between compliance and length of use of antiretrovirals (p=0.018), sexual orientation (p=0.024) and number of children (p=0.029).

**Conclusion:**

the coinfected patients presented inappropriate adherence to the antiretrovirals, a fact that negatively affects the health conditions of the people living with HIV/tuberculosis coinfection. A statistically significant correlation was found between the levels of adherence and some sociodemographic and clinical characteristics.

## Introduction

Infection by the Human Immunodeficiency Virus (HIV) has been considered one of the main risk factors for the development of active Tuberculosis (TB), based on a latent infection in people infected with *Mycobacterium tuberculosis* ^(^ 1 ^)^.

HIV infection represents a significant challenge for global TB control. TB is the second main cause of death by infectious diseases all over the world and the main cause among people living with HIV. Approximately 13.0% of TB cases happen in people living with HIV^(^ 2 ^)^.

Compliance with TB and HIV/AIDS treatment is fundamental to control these infections as, despite being considered chronic infection, tuberculosis treatment takes between six and nine months, depending on the type of TB, while HIV/AIDS treatment lasts a lifetime^(^ 3 ^)^.

HIV/TB coinfection results in higher mortality rates than HIV infection only^(^ 4 ^)^. Resistance to tuberculostatic drugs, as well as the high risk of transmitting the TB bacillus, emerged due to the abandonment and inappropriate use of drugs against TB. People living with HIV are at greater risk of reactivating the latent tuberculosis infection due to the deficient immune response. In coinfected people, mortality is commonly related with late diagnosis, as some people with HIV postpone visiting a health service out of fear of receiving an AIDS diagnosis^(^ 5 ^)^.

Concerning the disease evolution, the reduced survival of HIV/AIDS patients has been observed after the development of active TB. In addition, the HIV infection changes the TB infection, its clinical manifestation, the length of the treatment and tolerance of tuberculostatic drugs. As a result of correct treatment, however, approximately 90.0% of coinfected patients with active TB can be cured and discharged^(^ 6 ^)^.

Antiretroviral Therapy (ARVT) significantly reduces the risk of morbidity and mortality due to TB. A study appoints that the appropriate use of ARVT reduces the risk of developing TB by 65.0%, independently of the T CD4+ lymphocyte count^(^ 7 ^)^.

People being concomitantly treated for both diseases can be at risk of reduced adherence to one or both treatments. The low compliance with TB and HIV treatment can lead to the increased risk of drug resistance, relapses, death and, in addition, extend the infectiousness^(^ 8 ^)^.

The characteristics frequently appointed in research concerning adherence to medication related to different health conditions include patients' social, environmental, cultural and psychological aspects, as well as the characteristics intrinsic to the treatment^(^ 1 ^)^.

In view of the above, the goals were to assess the compliance levels with antiretroviral therapy in patients coinfected with HIV/tuberculosis and to correlate these levels with the sociodemographic and clinical variables of the study population.

## Method

A descriptive and cross-sectional study was undertaken at a public teaching and referral hospital for the diagnosis and treatment of infectious diseases in the State of Ceará. The data were collected in a consecutive period of six months, from September 2012 till January 2013.

The population consisted of 74 people coinfected with HIV/TB and monitored at the service's outpatient clinic, whose inclusion criteria were: age 18 years or older, male or female, with a medical diagnosis of HIV and tuberculosis (HIV/TB coinfection), formally registered in the patient history. This number represented 90.2% of all coinfected patients attended at the service during the research period.

The participants were recruited for the study while attending their routine outpatient consultation. The interviews were held at a private room, permitting the secrecy and confidentiality of the information obtained.

To collect the data, a sociodemographic and clinical assessment tool was used. The variables obtained from primary sources were: age, sex, self-referred color, education, occupation, income, marital situation, sexual orientation, number of children and number of people living in the same home. The clinical data were: current serum status of partner, length of diagnosis of HIV infection, length of use of T CD4+ lymphocyte counts, viral load and form of exposure to tuberculosis.

To assess the compliance with antiretroviral treatment, the Brazilian version of the Antiretroviral Treatment Adherence Assessment Questionnaire (CEAT-HIV) was used, a tool applicable to people infected with HIV, containing 20 questions, in which the total score is obtained by adding up all item scores (minimum possible score 17, maximum possible score 89). The higher the score, the higher the degree of treatment compliance. The degree of compliance follows these categories: inappropriate or low/insufficient (gross score ≤74), good/appropriate (gross score between 75 and 79) and strict (gross score ≥80)^(^ 9 ^)^. In this study, for the purpose of the data analysis, considering that Fisher's exact chi-square test was used, adherence was classified as good/appropriate and strict (gross score ≥75) and inappropriate or low/insufficient (gross score ≤74). Inappropriate adherence is characterized by complete treatment abandonment or incorrect follow-up.

For the statistical analyses, the software STATA version 11.0 was used. The sociodemographic and clinical characteristics were processed through descriptive statistics, uni and bivariate frequency distributions and descriptive measures (average and standard deviation). The p-values were obtained using Fisher's exact chi-square test and the probability test. Significance was set at 5.0% (p≤0.05).

The study received approval from the Research Ethics Committee under Protocol 93.437, complying with the recommendations of National Health Council Resolution 466/2012.

## Results

Among the 74 persons investigated, 79.7% were male. The predominant age range was between 30 and 39 years (35.1%), with a mean age of 37.7 years (standard deviation-sd=10.88), minimum age 20 years and maximum age 71 years.

In the distribution of the self-referred color, mulatto individuals were predominant (58.1%). As to the marital status, 31.1% were married or lived with a fixed partner. Concerning the sexual orientation, 61.7% indicated they were heterosexual. Regarding education, 42.4% reported they had finished secondary education, with an average of approximately 9.3 years of study, minimum 0 and maximum 20 years of study. What the professional situation is concerned, it was observed that 40.5% were unemployed and 75.7% gained an income of less than one minimum wage (Table 1).

With regard to medication adherence, for this study, only consent with the antiretroviral therapy was considered, as the literature does not present any validated tool to measure compliance with ARVT and tuberculostatic drugs at the same time among patients confected with HIV/TB.

In Table 1, the correlations between the sociodemographic characteristics and the levels of compliance with the antiretroviral drugs are observed among the patients coinfected with HIV/TB. In the total group, 10 (13.5%) were classified under appropriate adherence, and 64 (86.5%) presented inappropriate levels.

When the variables sex, age, color, marital situation, education, occupation, income and number of people living at the same home were assessed, correlated with the adherence levels, no statistically significant differences were observed. On the opposite, statistically significant proportional differences were obtained between sexual orientation and adherence (p=0.024) and number of children and adherence (p=0.029) (Table 1).

**Table 1:** - Distribution of sociodemographic characteristics of 74 patients coinfected with HIV/tuberculosis and compliance levels with antiretrovirals. Fortaleza, CE, Brazil, 2012

Variables	Adherence to ARVT*	Total	P	
	Appropriate N (%)	Inappropriate N (%)	N (%)	
Sex				0.676
	Male	9 (90.0)	50 (78.1)	59 (79.7)
	Female	1 (10.0)	14 (21.9)	15 (20.3)
Age (years)				0.22
	Up to 29	4 (40.0)	14 (21.9)	18 (24.3)
	30-39	3 (30.0)	23 (36.0)	26 (35.1)
	40-49	3 (30.0)	20 (31.2)	23 (31.1)
	50-59	0 (0.0)	3 (4.7)	3 (4.1)
	≥60	0 (0.0)	4 (6.2)	4 (5.4)
Self-referred color				1.000
	White	4 (40.0)	26 (40.6)	30 (40.5)
	Black	0 (0.0)	1 (2.5)	1 (1.4)
	Mulatto	6 (60.0)	37 (57.9)	43 (58.1)
Marital situation				0.768
	Married/Fixed partner	3 (30.0)	17 (26.5)	20 (27.0)
	Single	7 (70.0)	39 (61.0)	46 (62.2)
	Others	0 (0.0)	8 (12.5)	8 (10.8)
Sexual orientation				0.024
	Heterosexual	5 (50.0)	43 (67.2)	48 (64.9)
	Homosexual	5 (50.0)	9 (14.0)	14 (18.9)
	Bisexual	0 (0.0)	12 (18.8)	12 (16.2)
Education				0.21
	Illiterate	0 (0.0)	6 (9.3)	6 (8.1)
	Primary education	2 (20.0)	30 (46.9)	32 (43.2)
	Secondary education	7 (70.0)	28 (43.8)	35 (47.3)
	Higher education	1 (10.0)	0 (0.0)	1 (1.4)
Occupation				0.828
	Employed	3 (30.0)	13 (20.3)	16 (21.6)
	Unemployed	4 (40.0)	26 (40.7)	30 (40.5)
	Others	3 (30.0)	25 (39.0)	28 (37.9)
Income (minimum wage)†				0.672
	<1	7 (70.0)	49 (76.5)	56 (75.7)
	1-2	2 (20.0)	12 (18.7)	14 (18.9)
	>2	1 (10.0)	3 (4.69)	4 (5.4)
Number of children				0.029
	0	8 (80.0)	22 (34.4)	30 (40.6)
	1-2	1 (10.0)	21 (32.8)	22 (29.7)
	≥3	1 (10.0)	21 (32.8)	22 (29.7)
Number of people living per home				0.421
	1	0 (0.0)	11 (17.1)	11 (14.9)
	2-3	5 (50.0)	21 (32.8)	26 (35.2)
	≥4	5 (50.0)	32 (50.0)	37 (50.0)

*Antiretroviral therapy; †Minimum wage in 2012 in Brazil: R$622.00.

When assessing the HIV/TB coinfected patients' clinical characteristics and the correlation with the level of adherence, it was observed that, concerning knowledge of the sexual partner's serum status of the 23 people whose partners took the anti-HIV test, adherence was inappropriate in 86.9% (n=20), independently of the test result. The three people with appropriate adherence had a seroconcordant current sexual partner (Table 2).

Statistical significance was obtained for the length of use of ARVT and compliance levels (p=0.018), but the other variables (serum status of partner, length of diagnosis, CD4+ count, viral load and hospitalizations) showed no proportional similarity, as the p-values were higher than 0.05 (p≥0.05).

**Table 2:** - Distribution of clinical characteristics of 74 patients coinfected with HIV/tuberculosis and compliance levels with antiretrovirals. Fortaleza, CE, Brazil, 2012

Variables	Compliance with ARVT *	Total	p	
	Appropriate N (%)	Inappropriate N (%)	N (%)	
Serum status of partner (n=23)				1.000
	Positive (n=16)	3 (100.0)	13 (65.0)	16 (69.7)
	Negative (n=3)	0 (0.0)	3 (15.0)	3 (13.0)
	Does not know/not tested (n=4)	0 (0.0)	4 (20.0)	4 (17.4)
Length of HIV diagnosis (years)				0.680
	<1	6 (60.0)	28 (43.8)	34 (46.9)
	1-5	3 (30.0)	22 (34.3)	25 (33.8)
	>5	1 (10.0)	14 (21.9)	15 (20.3)
T CD4+ lymphocytes (cells/mm³)				0.528
	<200	7 (70.0)	31 (48.5)	38 (51.4)
	200-499	3 (30.0)	29 (45.3)	32 (43.2)
	≥500	0 (0.0)	4 (6.2)	4 (5.4)
Viral load (copies/ml)				0.190
	<50	5 (50.0)	14 (22.0)	19 (25.7)
	50-10.000	1 (10.0)	9 (14.0)	10 (13.5)
	>10.000	4 (40.0)	41 (64.0)	45 (60.8)
Length of use of ARVT (months)				0.018
	<6	2 (20.0)	34 (53.1)	36 (48.6)
	6-12	5 (50.0)	8 (12.5)	13 (17.6)
	>12	3 (30.0)	22 (34.4)	25 (33.8)
Hospitalizations due to complications of HIV				0.749
	0	4 (40.0)	18 (28.1)	22 (29.7)
	1-2	4 (40.0)	33 (51.5)	37 (50.0)
	>3	2 (20.0)	13 (20.4)	15 (20.3)

*Antiretroviral therapy.

When analyzing the forms of exposure to tuberculosis, the predominance of the pulmonary form was demonstrated and, concerning the correlation with adherence to ARVT, no statistically significant difference was observed (p=0.374) (Table 3).

**Table 3:** - Distribution of tuberculosis presentation forms and levels of compliance with antiretrovirals of 74 patients coinfected with HIV/tuberculosis. Fortaleza, CE, Brazil, 2012

Type of tuberculosis	Compliance with ARVT *	Total	P	
	Appropriate N (%)	Inappropriate N (%)	N (%)	
Pulmonary	6 (60.0)	47 (73.4)	53 (71.6)	0.374
Extrapulmonary	4 (40.0)	13 (20.3)	17 (22.9)	
Pulmonary + extrapulmonary (mixed)	0 (0.0)	4 (6.3)	4 (5.4)	

*Antiretroviral therapy.

## Discussion

Similar to the present findings, the HIV/TB coinfection was more frequent in individuals between 30 and 39 years of age, representing the economically most active part of the population^(^ 10 ^-^ 11 ^)^. In Brazil, the AIDS epidemic has grown among younger individuals and women. As observed in other studies^(^ 11 ^-^ 12 ^)^, however, the population with HIV/TB coinfection predominantly included men of economically productive age.

Among the study variables, education is strongly associated with precarious socioeconomic conditions. Using it as a substitute measure of poverty, this suggests that people with this coinfection are in a situation of impoverishment, with a reduced ability to cope with the consequences of diseases due to the deficient access to preventive, diagnostic and curative services^(^ 13 ^)^.

A significant association was reported between HIV/TB coinfection and living in an urban area and extrapulmonary TB. After the establishment of AIDS, it is known that the extrapulmonary forms of TB become more common^(^ 10 ^)^. Concerning the form of TB, this information differs from the present study findings, in which pulmonary tuberculosis was the predominant clinical form in people living with HIV.

As regards the occupational situation, there are high unemployment rates among coinfected patients. This finding was also observed in this study, demonstrating that the economic and social difficulties can influence their professional possibilities, maintaining their conditions of poverty^(^ 14 ^)^.

In another study, the professional activity was mentioned as one of the most relevant factors to cope with the condition of the HIV infection. The work environment was appointed as a place of contact and experience exchange among people, favoring mental coping with the infection. In the disease situation, the availability of social support raises the self-esteem and the desire to live, contributing to the success of their treatment^(^ 15 ^)^.

The participants in this study presented low T CD4+ cell counts and a high viral load, supporting the literature and indicating that TB is an opportunistic infection, strongly associated with the decrease of the immunological system. It is observed that, overall, people coinfected with HIV/TB present greater immune system problems when compared to people who simply suffer from HIV infection in the asymptomatic phase^(^ 12 ^)^.

The most frequent difficulties people living with HIV/TB coinfection face are related to socioeconomic aspects, social factors, lifestyle and difficulties with the therapeutic regimens and with the intervals between the drug doses, characterized as motives that interfere significantly in continuing and effective adherence^(^ 16 ^)^.

The treatment of coinfected people tends to be difficult. TB patients need long-term treatment with different drugs. For HIV/TB patients, compliance with the therapeutic scheme is difficult as they have to use concomitant drugs to treat both infections^(^ 10 ^)^. This situation may have contributed to the predominantly inappropriate compliance with ARVT in this study.

A study indicates that the variables significantly associated with non-compliance with the double therapy were: male sex, low income, presence of three or more chronic conditions, having a knowingly HIV-positive partner and sexual relationships in the previous three months^(^ 1 ^)^.

In a study aimed at identifying the determinants of medication compliance in patients coinfected with HIV/TB, it was demonstrated that a large number of people living at the same home, lack of financial resources to promote self-care and deficient knowledge on the diseases were factors that interfered in appropriate adherence to the medication. The sociodemographic variables were not considered as predictive factors that influenced medication adherence^(^ 3 ^)^, differently from the scientific hypothesis formulated for this study.

In the assessment of the quality of life of people living with HIV/AIDS, according to the levels of compliance with the antiretroviral drugs, it was identified that the individuals classified and non-compliant with the treatment had worse scores on all quality of life domains, demonstrating that non-compliance with the treatment is a widespread problem, negatively affecting patients and the population in general^(^ 17 ^)^.

## Conclusion

Compliance with ARVT among the coinfected patients was inappropriate in a significant part of patients on antiretroviral drugs. In addition, a statistically significant difference was observed between the levels of compliance with the antiretroviral drugs and the length of use of ARVT, sexual orientation and the number of children.

The data demonstrated implications related to medication adherence and double infection, whose appropriate intake of the drugs is needed to achieve better clinical and health conditions. In addition, the consequences of the lack of compliance particularly affect the patients' survival and can cause higher mortality rates among people living with HIV/TB coinfection.

This study appoints the need to develop a specific compliance measure for people coinfected with HIV/TB, thus demonstrating a limitation in the assessment of coinfected patients' compliance, as the analysis was restricted to compliance with ARVT only.
